# A case report of persistent risky dietary behaviors in a bipolar disorder patient

**DOI:** 10.1186/s12888-019-2335-9

**Published:** 2019-11-08

**Authors:** Yanping Duan, Jinya Cao, Paul Summergrad, Jing Wei

**Affiliations:** 1 Department of Psychological Medicine, Peking Union Medical College Hospital, Chinese Academy of Medical Science and Peking Union Medical College, Beijing, 100730 China; 2 Psychiatry and Inflammation Program, Department of Psychiatry, Tufts University School of Medicine, Boston, MA USA

**Keywords:** Bipolar disorder, Dietary behavior, Risk-taking behavior, Parasitic infections, Organic mental disorder

## Abstract

**Background:**

Bipolar disorder is associated with complicated medical comorbidities. The risk-taking behavior of bipolar disorder patients may lead to many problems.

**Case presentation:**

A 40-year-old male patient had gastrointestinal symptoms for 4 months. He was talkative, agitative, and grandiose but showed poor cognition. Multisystem injury required multidepartment, multidisciplinary consultation. Repeated fecal examination found multiple infections of Opisthorchis sinensis, Heterophyes, and Echinostomatidae. The diagnostic criteria for parasitic infections, bipolar disorder and organic mental disorder were met. After treatment with a mood stabilizer and helminthic, his mood became stable, but risky dietary behavior continued.

**Conclusions:**

The case describes persistent risky dietary behaviors in a bipolar patient even after affective symptoms were under control, which ultimately led to diverse parasitic infections and chronic encephalopathy. We call for clinical and scientific attention to possible dangerous behavior changes in bipolar patients even after their emotions are stabilized.

## Background

Bipolar disorder is a common affective disorder that includes depressive episodes and manic/hypomanic episodes and is estimated to affect 2 to 5% of the population [1]. Bipolar disorder may be due to physiopathological mechanisms involving molecular biology, neuroanatomy, genetics, or medical conditions [2]. Bipolar disorder is associated with complicated medical comorbidities and an increased risk of death from chronic medical illness [3]. As described by Krauthamer and Klerman, mania can occur in association with organic dysfunction in patients with no history of affective disorder secondary to drugs, infection, neoplasm, epilepsy, and metabolic disturbances [4]. Alternatively, one of the consequences of bipolar disorder may be changes in risk-taking behaviors. We present a case of bipolar disorder in which the persistent risky dietary behavior that continued may have led to diverse parasitic infections with multiple organ system dysfunctions.

## Case presentation

A 40-year-old male patient complained of diarrhea and poor appetite for 4 months and fatigue and lower limb edema for 3 months.

### History of presenting complaint

His earliest symptoms were gastrointestinal: anorexia, nausea, vomiting of green stomach contents, and green-colored diarrhea. Later, symptoms of cardiac dysfunction, such as edema, fatigue and decreased activity, were found. An ultrasonic electrocardiogram at the local hospital suggested that a 25 mm*22 mm space-occupying lesion was found in the posterior lobe of the left atrium near the mitral valve, and repeated examinations showed that the occupation gradually increased to 68 mm*48 mm. Chest computed tomography (CT) showed a right upper lung cavity and a double lung plaque, and sputum culture suggested fungal infection. He was taken to the emergency department of Peking Union Medical College Hospital for multiple system injuries.

### Diagnostic symptoms

**Fig. 1.**
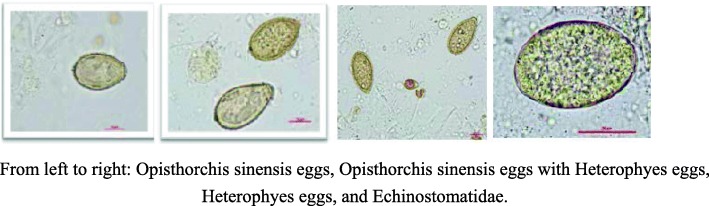
Parasite eggs observed under high magnification of a fecal smear using a microscope. From left to right: Opisthorchis sinensis eggs, Opisthorchis sinensis eggs with Heterophyes eggs, Heterophyes eggs, and Echinostomatidae

**Parasitic infections**: After repeated microscopic examinations of his feces, Opisthorchis sinensis eggs, Heterophyes eggs, and Echinostomatidae were found (Fig. 1). Opisthorchis sinensis can migrate in the body. In cardiac surgery, doctors found a perforation of the mitral valve. Intracardiac vegetation was thought to be related to Aspergillus infection, and parasitic infections facilitated the Aspergillus infection. However, no parasites were found in the limited myocardial tissue with histopathological examinations. Opisthorchis sinensis infection in the brain may cause convulsions and paralysis [5]. The brain MRI of the patient showed chronic infarction. Brain magnetic resonance imaging (MRI) of the patient showed chronic infarction, which may be the result of parasite immune evasion [6]. Because the damaged brain areas were functionally active areas, no biopsy could be undertaken to verify whether there were parasites. **Bipolar disorder**: The patient was talkative, agitative, grandiose, and overactive. He had a possible episode of depression after his father passed away and two manic episodes, which were diagnosed by local psychiatrists, with symptoms including elation, talkativeness, feeling energetic, reduced need for sleep, increased activity, dangerous driving, and risky dietary behavior during 2009 and 2013. A timeline of the history is shown in graph 4.

### Differential diagnostic symptoms

Gastrointestinal symptoms suggested parasitic infections. Talkativeness, grandiosity and overactive symptoms suggested bipolar disorder. Multiorganic injuries, poor cognition and persistent behaviors might not be interpreted as bipolar disorders. **Dementia:** The patient had poor cognition, confabulation and euphoria, which could be interpreted as chronic encephalopathy. The patient had diarrhea and seizures for 4 months, so the clinically reasonable deduction was that his encephalopathy had developed in the last several months. However, he had been eating raw food with a parasitic infection risk for 8 years, so his changes in eating behavior could not be explained by dementia. **Delirium:** Delirium is defined as nonspecific acute encephalopathy syndrome with consciousness, attention, thinking, memory, mental motor behavior, and sleep cycle disorder [7]. Delirium is usually transient, lasting for a maximum of 4 weeks and persisting for 6 months in only a few patients. This patient had severe and complex physical diseases, cardiac surgery, and mental disorders, which made the patient prone to delirium. However, after his physical condition stabilized during follow-up, the patient still had poor cognition and euphoria. This is better explained by chronic encephalopathy rather than delirium. However, the agitation described during the first consultation may have partly been caused by delirium. **Klüver-Bucy syndrome**: Heinrich Klüver and Paul Bucy described a dramatic behavioral syndrome that includes hyperorality, placidity, hypermetamorphosis, dietary changes, altered sexual behavior, and visual agnosia, in monkeys after bilateral temporal lobectomy in 1937. It is now thought to be caused by disturbances of the temporal portions of the limbic networks that interface with multiple cortical and subcortical circuits to modulate emotional behavior and affect [8]. Subcortical infarction and inflammation of the brain might cause Klüver-Bucy syndrome, which might support episodes of docility but not hyperactivity. **Schizoaffective disorder:** The patient had delusions of grandeur and affective symptoms; however, no sufficient symptoms met the diagnostic criteria for schizophrenia.

### Complications

**Fig. 2:**
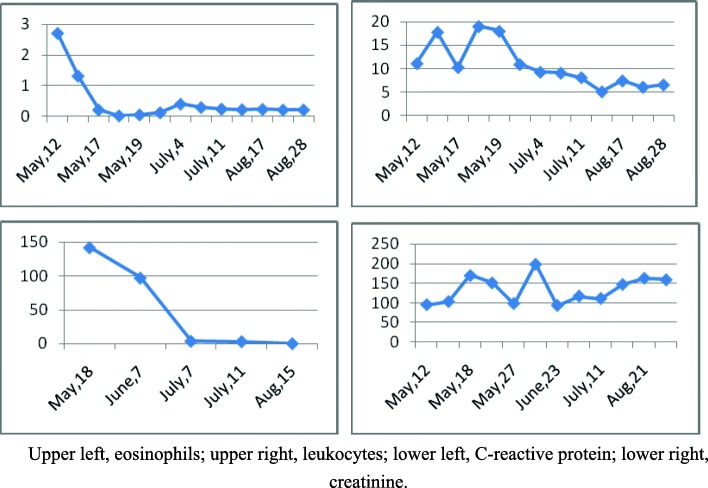
Changes to the blood tests of the patient during treatment. Upper left, eosinophils; upper right, leukocytes; lower left, C-reactive protein; lower right, creatinine

**Fig. 3:**
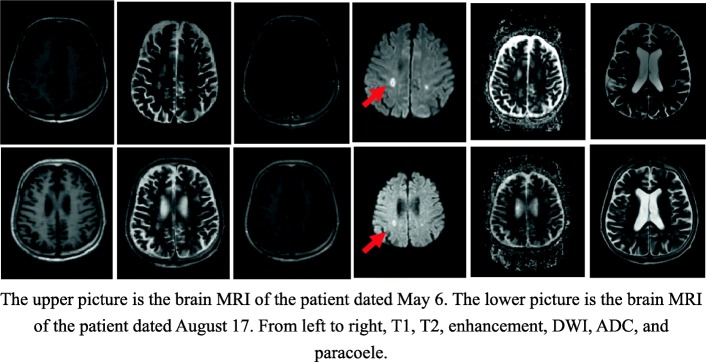
CNS MRI before treatment (above) and after treatment (below). The upper picture is the brain MRI of the patient dated May 6. The lower picture is the brain MRI of the patient dated August 17. From left to right, T1, T2, enhancement, DWI, ADC, and paracoele

Aspergillus was found in the patient’s blood. His eosinophil and leukocyte counts as well as his C-reactive protein and blood creatinine levels were also abnormal (Fig. 2). The patient had three episodes of seizures during hospitalization. A central nervous system (CNS) MRI was taken before the surgery and 2 months thereafter (Fig. 3). Subcortical infarctions were found in the bilateral centrum semiovale and the left ventricular posterior horn. His left cerebellar hemisphere showed chronic infarction. Ischemic changes of white matter in the bilateral frontal and parietal lobe were detected, and abnormal signals in ventriculus lateralis cerebri could be found.

### Causes/etiology

**Fig. 4:**
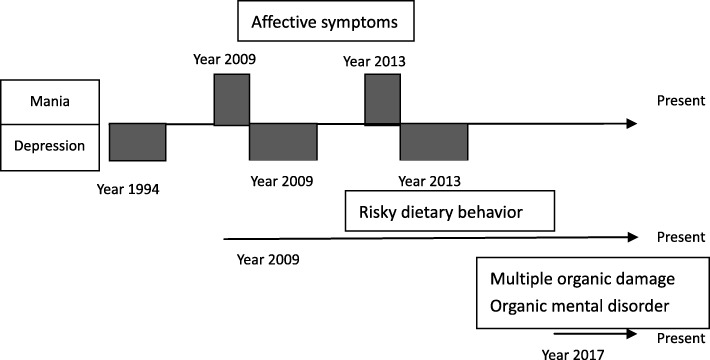
Medical history of the patient

A timeline of the history is shown in Fig. 4. Risky dietary behavior started during a manic episode and persisted even after affective symptoms were under control. Risky dietary behavior might have caused parasitic infection, which led to multiple organic injuries.

### Past psychiatric history

The medical history was obtained from the patient’s mother and his sister. His father died in 1994. The patient was depressed for several months, nontalkative, sleepless, and had no appetite. Nevertheless, he recovered spontaneously. During 2009, he suddenly developed a high level of energy, required very little sleep, and undertook risky and dangerous behavior, including in his manner of driving. After a brief period, he abruptly became depressed. “Depression of manic-depressive disorder” was diagnosed by his local psychiatrists, but details of his psychopharmacologic treatment could not be provided by his mother. He stopped his prescribed medication when his symptoms were relieved. His dietary behavior changed permanently since 2009. He continued to eat raw ox-gall, snake gall, frog, and tadpole. During 2013, similar emotional symptoms reoccurred. After a few days of hyperactivity, he suddenly became depressed and very dependent on his mother’s company. He ceased working and stayed in his seaside house alone (Fig. 4).

### Past medical and surgical history

The patient suffered from diabetes mellitus for 10 years. He had no surgical history.

### Family and social history

The patient did not have a family history of psychiatric disorders.

### Personal and forensic history

The patient came from Shandong Province in China. He was unmarried and living alone beside the seashore. He was a civil servant. He had stayed at home on leave for 8 years because of bipolar disorder.

### Mental status examination

The patient had full orientation. He was euphoric, talkative, charismatic, and grandiose. He was agitated when he was interrupted. Sometimes he suddenly became angry and refused to continue the conversation. He had no insight into his disease. The total score on the MMSE (mini-mental state examination) was 24; he made errors in execution, reading, and writing. His appetite was unusually high. He frequently expressed hunger, and he ate voraciously. He expressed sexual interest, repetitively saying “I want to marry all the nurses in the hospital.”

### Follow up/reviews

After treatment with Debagin 500 mg per day, olanzapine 5 mg per day and praziquantel 210 mg/kg three times a day for 3 days, the patient’s mood improved, and his physical condition was restored. However, he was still talkative and still showed eagerness for sex and raw food. Three months later, his sister said that the patient had stopped all medication since leaving the hospital. He showed overeating, and his body weight increased. He became quiet unless spoken to, but there were no significant changes in his brain MRI (graph 2). Six months later, he was still eager to eat raw food with soy sauce, but his family no longer allowed him to eat tadpoles. He became quiet but still demonstrated confabulation and grandiose symptoms. At the one-year follow-up, he still demonstrated poor cognition and confabulation. His sister said that the patient was quiet at home. He was still fond of barbecue and believed that raw food with soy sauce was delicious. However, his family restricted him from raw food, such as tadpoles and frogs.

## Discussion and conclusions

This case describes persistent risky dietary behaviors in a patient with bipolar disorder even after affective symptoms were under control, which ultimately led to diverse parasitic infections and chronic encephalopathy. A limitation of this case is that it was difficult to collect the patient’s medical history. The patient’s medical history could only be confirmed by his family members.

It is extremely rare to find three types of parasites in one person. Opisthorchis sinensis is common in some provinces of China. A nationwide survey conducted from 2001 to 2004 showed that the prevalence of Opisthorchis sinensis ranged from 0.31 to 16.4% [9]. The investigation of frogs in Dezhou of Shandong showed that the infection rate of Opisthorchis sinensis was 2.86% [10]. The patient may have become infected with Opisthorchis sinensis by eating raw frogs. Heterophyid trematodes are found in Taiwan but have seldom been found in mainland China [11]. Echinostomatidae has been found in Guangdong Province in China and has been more rarely reported in Shandong Province [12]. Given the highly developed goods transfer network in China, it should not be difficult to obtain food that has been transported from any part of China.

How can his risky dietary behaviors be understood? It is possible that these behaviors occurred because of the bipolar disorder. Risk-taking behavior can be a key component of bipolar disorder. Disinhibition of eating and increased perception of hunger have been reported in patients with bipolar disorder [13]. These abnormal eating behaviors can lead to obesity. In our case, the patient had malnutrition instead of obesity, and his eating preferences were raw tadpoles and frogs, which he did not consider risk-taking behavior. Eating raw but professionally prepared and checked seafood, such as salmon and oyster, occurs in seashore cities, such as Qingdao in China, in Japan and throughout Europe and the United States. However, the fish undergoes careful inspection and, in some cases, flash freezing. Eating raw frogs and tadpoles is bizarre and disturbing according to any Chinese dietary culture. The patient was not just unaware when choosing a restaurant or chef; his behavior was well outside the normal standard of Chinese dietary culture. Our hypothesis is that this behavior may have been a risk-taking behavior when he felt that he was powerful and had uncontrolled impulses and when he ignored the risks of dangerous behaviors. What is interesting in this case is that this behavior persisted even when he was not in an elated mood. Other manic patients may show similar behavior changes. Additionally, when patients are in an emotionally stable state, they are outside the scope of clinical observation.

Risk-taking behaviors, such as dangerous driving, unsafe sexual conduct and hasty financial decisions, are common in patients with bipolar disorder [14]. However, the desire for food, which is part of human nature, is given little attention, especially regarding what and how to eat. In our case, dietary preferences and binge-eating behavior may have increased the risk of multiple parasitic infections, which resulted in serious organic lesions.

Risky dietary behaviors in a bipolar patient may persist after affective symptoms are under control, which may lead to diverse parasitic infections and chronic encephalopathy. Conversely, Klüver-Bucy syndrome may cause strange behavior, including bizarre eating behavior and persistent eroticism. Therefore, we call for clinical and scientific attention by psychiatrists to possible dangerous behavior changes among bipolar patients even after their emotions are stabilized.

## Data Availability

Not applicable.

## Abbreviations

BMI: Body mass index
CNS: Central nervous system
CT: Computed tomography
ICD-10: The international classification of diseases
MMSE: Mini-mental state examination
MRI: Magnetic resonance imaging
